# Two-phase simulation of entropy optimized mixed convection flow of two different shear-thinning nanomaterials in thermal and mass diffusion systems with Lorentz forces

**DOI:** 10.1038/s41598-023-50725-w

**Published:** 2024-01-04

**Authors:** S. Suresha, Umair Khan, D. O. Soumya, P. Venkatesh, Hatem Gasmi, M. Sunitha, Aurang Zaib, Ahmed Al-Naghi, Hatem Karoui, Anuar Ishak, Walter Ojok

**Affiliations:** 1 Department of Physics, Government First Grade College, Santhebennur, 577552 India; 2 Department of Mathematical Sciences, Faculty of Science and Technology, Universiti Kebangsaan Malaysia, UKM, Bangi, 43600 Selangor Malaysia; 3 Department of Computer Science and Mathematics, Lebanese American University, Byblos, 1401 Lebanon; 4 Department of Mathematics and Social Sciences, Sukkur IBA University, Sukkur, 65200 Sindh Pakistan; 5 Department of Mathematics, PES Institute of Technology & Management, Shivamogga, Karnataka India; 6 Department of Mathematics, Sahyadri Science College, Shivamogga, Karnataka India; 7 Department of Civil Engineering, College of Engineering, University of Hail, Hail, Saudi Arabia; 8 LR14ES03 Laboratoire d’Inge´nierie Ge´otechnique, Ecole Nationale d’Inge´nieurs de Tunis, Universite´ de Tunis El Manar, 1002 Tunis, Tunisia; 9 Department of Mathematics and Statistics, University College for Women Koti, Hyderabad, India; 10 Department of Mathematical Sciences, Federal Urdu University of Arts, Science & Technology, Gulshan-E-Iqbal, Karachi, 75300 Pakistan; 11 Department of Chemistry, Faculty of Science, Muni University, P.O Box 725, Arua, Uganda

**Keywords:** Energy science and technology, Engineering, Mathematics and computing, Nanoscience and technology

## Abstract

This research compares the momentum, thermal energy, mass diffusion and entropy generation of two shear thinning nanofluids in an angled micro-channel with mixed convection, nonlinear thermal radiation, temperature jump boundary condition and variable thermal conductivity effects. The $RKF 45$ approach was used to solve the Buongiorno nonlinear governing model. The effect of different parameters on the flow, energy, concentration, and entropy generating fields have been graphically illustrated and explained. The hyperbolic tangent nanoliquid has a better velocity than the Williamson nanofluid. The Williamson nanofluid has higher thermal energy and concentration than the hyperbolic tangent nanoliquid in the microchannel. The Grashof number, both thermal and solutal, increases the fluid flow rate throughout the flow system. The energy of the nanoliquid is reduced by the temperature jump condition, while the energy field of the nanoliquid is enhanced by the improving thermal conductivity value. The nanoliquids concentration rises as the Schmitt number rises. The irreversibility rate of the channel system is maximized by the variable thermal conductivity parameter.

## Introduction

Fluids whose flow behavior is not well explained by Newton’s law of viscosity are referred to as non-Newtonian liquids. Their viscosity shifts in response to changes in shear rate, time, pressure, or temperature, in contrast to the behavior of Newtonian liquids. There are many different kinds of non-Newtonian liquids like Casson^1–4^, second grade^5^, third grade^6^, fourth grade^7^ micropolar^8^, Burgers fluid^9,10^ and Maxwell fluids^11,12^. Some of these fluids are viscoelastic, while others are shear-thickening. Non-Newtonian fluid models include the Williamson and hyperbolic tangent fluid models. These are the fundamental models for modeling the viscoelastic shear thinning properties of non-Newtonian fluids. These shear thinning fluid models are widely employed in a variety of scientific studies. Thermal conductivity of nanofluid has been shown to be a desirable property for a variety of applications. It refers to a substance that may either conduct or transmit heat. The channel flow has been analyzed with great interest by numerous researchers for shear thinning fluids because of many of applications. Khan et al.^13^ considered a curved moving surface to scrutinize the thermal and flow properties of Williamson fluid along with magnetic field. Mahdy^14^ studied the flow behavior of tangent-hyperbolic-fluid in a stretched cylinder numerically. They revealed that the temperature of the fluid rises as the curvature parameter rises. To explore mass and heat transmission in the shear thinning fluid, Ghulam et al.^15^ used the Buongiorno model. They consider stretching non-linear surface to scrutinize the fluid's flow characteristics. The cross flow and thermal characteristics of the Williamson fluid in a shrinking/stretching geometry with radiative effects were studied by Khan et al.^16^. The entire investigation was done using a dual solution. Ashraf et al.^17^ explored characteristics of tangent hyperbolic fluid on a slender surface with bioconvection. Zhou et al.^18^ investigated the convective Williamson nanoliquid flow phenomenon with gyrotactic microorganism suspension.

Nanofluids are a kind of heat transfer fluid made up of a base fluid and nanoparticles, which are generally metallic or non-metallic particles with diameters ranging from 1 to 100 nm. Nanoparticles are added to the base liquid to improve its thermophysical characteristics, making nanoliquids a viable technology for a variety of heat transport applications. Recently, the water-based nanoliquid motion via parallel fins contained within a partly heated hexagonal chamber was investigated by Acharya and Chamkha^19^. Acharya^20–23^ studied the hydrothermal behavior of different liquids with suspension of mono, hybrid and tri hybrid nanoparticles past diverse surfaces by considering various physical aspects. Thermal increase in buoyancy-driven stagnation point motion of ternary nanoliquid across an inclined porous cylinder with radiation was inspected by Adnan et al.^24^. Hussain et al.^25^ explored the unsteady convection–diffusion transport issue.

The strength and direction of a uniform magnetic field are the same at all places. The collections of parallel lines with equal spacing are used to symbolize it. Lou et al.^26^ consider the aligned magnetic force to investigate the temperature and flow properties of the dusty micropolar fluid in a rotating surface. Norzawary et al.^27^ investigated the heat transfer properties of suspended hybrid nanofluid by the applications of micropolar fluid. Their results showed the multiple solutions for the case of shrinking. By applying Riga plate, Eswaramoorthi et al.^28^ applied Darcy law to investigate the non-Newtonian fluid which flows in laminar form. Upadhya et al.^29^ scrutinized the impact of Lorentz strength on the temperature and flow properties of hybrid nanofluids. In this work they revealed that the flow rate is the decreasing function of Lorentz force. Wang et al.^30^ scrutinized the impact of uniform magnetic field on the temperature and flow properties of hybrid nanofluids.

The ability to flow makes a liquid appropriate for eliminating excess heat from mechanical components. Liquids have better thermal conductivity than gases. The conductivity of non-metallic liquids diminishes as the temperature rises. The flow of axisymmetric ternary hybrid nanofluids in a surface with variable thermal conductivity properties has been inspected by Raju et al.^31^. By using a variable thermal conductivity property, Usha et al.^32^ examined the heat transfer and flow properties of an unsteady shear thinning fluid in the sensor surface. Gbadeyan et al.^33^ used the variable properties of the Casson nanofluid like, thermal conductivity and viscosity to inspect the momentum and thermal energy profile in flat vertical plate. The velocity profile of shear thinning fluid reduces with variable viscosity has been inspected by Salahuddin et al.^34^. Kumar et al.^35^ provided further details on the thermal distribution and effectiveness of a longitudinal rectangular fin that varies exponentially.

The thermal motion of molecules drives diffusion. At temperatures higher than absolute zero, molecules are never at rest. Distillation, drying, evaporation, alcohol distillation, and blood purification in the kidney are all examples of mass transfer processes. Srilatha et al.^36^ used the thermophoretic diffusion diffusion aspect to study the nanoliquid past an extended surface. The effect of a volume fraction on the concentration field of a nanofluid in a stretchy surface was discovered by Algehyne et al.^37^. The thermophoretic parameter is used to degrade the concentration fields. Zhao et al.^38^ used mass convective scenarios to investigate the diffusion mechanism in the ferromagnetic nanoliquid. The nanofluid behaviour in a channel was explored by Shilpa et al.^39^ under the influence of thermophoresis particle deposition mechanisms. The nanofluid behaviour in a gyrating disk was explored by Gowda et al.^40^ under the Brownian motion mechanisms.

Several major irreversible processes in a microchannel include fluid viscosity and diffusion. Mkwizu et al.^41^ examined the entropy generation rate at channel walls and discovered that the entropy generation rate of $Cu-{H}_{2} O$ and $A{l}_{2} {O}_{3}-{H}_{2}O$ nanoliquids differs at channel plates. $Cu-{H}_{2}O$ nanofluid has a lesser irreversibility rate at the bottom channel wall, while $A{l}_{2} {O}_{3}-{H}_{2}O$ nanoliquid has a lower rate at the above channel wall. The parameter of Casson fluid enhances the irreversibility rate in the channel system have been analyzed by Venkateswara et al.^42^. Using hybrid nanoliquid, Abderrahmane et al.^43^ take into consideration the convection flow to scrutinize the system's entropy generation. Nandi et al.^44^ demonstrated that by lowering the fluid flow rate, the porosity parameter improves the amount of irreversibility. The volume fraction enhances the horizontal poignant thin needle have been analyzed by Iqbal et al.^45^. Recently, numerous researchers explored the flow of different liquids with entropy generation^46–51^.

A deep insight into the literature review indicates the relatively few studies have been conducted on the entropy generation of shear thinning fluid utilizing Buongiorno model under the consequence of temperature jump boundary condition in the microchannel. Many applications in the mining, chemical and plastic processing industries require studying mass and heat transfer with Lorentz strength and varying thermal conductivity. The novelty of this study is that it compares the flow, thermal, and diffusion characteristics of two different shear thinning nanofluids in a microchannel with variable thermal conductivity, nonlinear thermal radiation, temperature jump boundary condition, solutal, and mass buoyancy force. In this paper, the following pertinent research questions were addressed:

- What is the impact of dimensionless parameters on flow, mass and heat transfer of Williamson and tangent hyperbolic nanoliquids?
- What is the impact of dimensionless parameters on entropy generation and Bejan number of Williamson and tangent hyperbolic nanoliquids.

## Mathematical modelling

**Figure 1:**
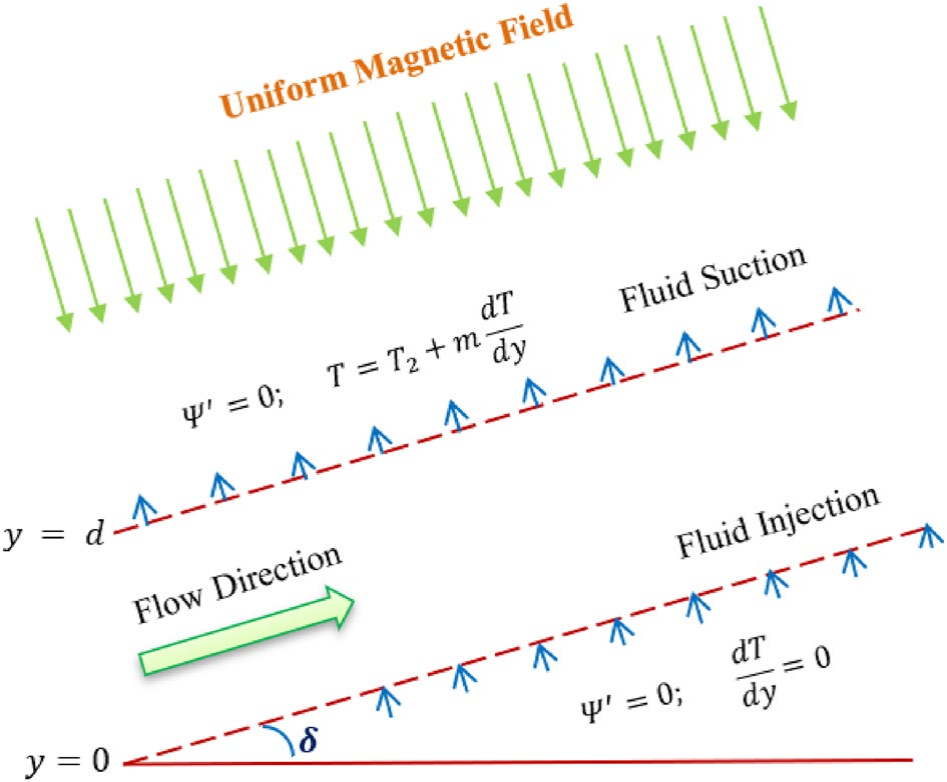
Physical model of the problem.

The schematic diagram of the flow configuration is shown in Fig. 1, where the experiment took into account the laminar flow of both Williamson and hyperbolic tangent nanofluid through a sloped microchannel, separately. The channel is formed by two permeable identical plates, which are separated by a distance 'd'. The Buongiorno model is used here and it illustrates the role of two significant mechanisms: Brownian motion and thermophoresis. By developing a mathematical flow model, the effects of Ohmic heating, viscous dissipation, temperature jump boundary, nonlinear thermal radiation and variable thermal conductivity are investigated. The magnetic flux is applied in a direction that is transverse to the flow direction. The injected hot liquid from the below porous channel plates heats the plate to temperature ${T}_{2},$ and the channel exchanges thermal energy with the surrounding of temperature ${T}_{1}$ by the suction process at the above channel plate. This investigation takes into account fully developed flow assumptions. The effect of buoyant force and chemical reaction are also considered in the analysis.

The equations that demonstrate the above-mentioned flow of Williamson nanofluid are as follows (see Ghulam et al.^15^, and Ashraf et al.^17^)

1 $$\rho {\nu }_{0}\frac{d{\Psi }^{\prime}}{dy}=-\frac{dp}{dx}+\mu \left[1+\sqrt{2} \Gamma \frac{d{\Psi }^{\prime}}{dy}\right]\frac{{d}^{2}{\Psi }^{\prime}}{d{y}^{2}}+\rho g\left[{\beta }_{T}\left(T-{T}_{1}\right)+\rho g{\beta }_{C}\left(C-{C}_{w}\right)\right]sinsin \left(\alpha \right)-\sigma {B}_{0}^{2}{\Psi }^{\prime},$$

2 $${\left(\rho {C}_{p}\right)}_{f} {\nu }_{0}\frac{dT}{dy}=\frac{d}{dy}\left({k}^{\mathrm{^{\prime}}}\frac{dT}{dy}\right)+\mu \left[1+\left(\frac{\Gamma }{\sqrt{2}}\right)\frac{d{\Psi }^{\mathrm{^{\prime}}}}{dy}\right] {\left(\frac{d{\Psi }^{\mathrm{^{\prime}}}}{dy}\right)}^{2}-\frac{d{q}_{r}}{dy}+\sigma {B}_{0}^{2}{{\Psi }^{\mathrm{^{\prime}}}}^{2}+{\left(\rho {C}_{p}\right)}_{p}\left[{D}_{B}\frac{dC}{dy}+\frac{{D}_{T}}{{T}_{1}}\frac{dT}{dy}\right]\frac{dT}{dy},$$

3 $${\vartheta }_{0 }\frac{dC\left(y\right)}{dy}-{D}_{B}\frac{{d}^{2}C\left(y\right)}{d{y}^{2}}-\frac{{D}_{T}}{{T}_{1}}\frac{{d}^{2}T\left(y\right)}{d{y}^{2}}+{\chi }^{\prime}\left(C\left(y\right)-{C}_{w}\left(y\right)\right)=0,$$

The following are the equations that exhibit the flow of hyperbolic tangent nanofluid stated above.

4 $$\rho {\nu }_{0}\frac{d{\Psi }^{\prime}}{dy}=-\frac{dp}{dx}+\mu \left[\left(1-n\right)+\sqrt{2} n \Gamma \frac{d{\Psi }^{\prime}}{dy}\right]\frac{{d}^{2}{\Psi }^{\prime}}{d{y}^{2}}+\rho g\left[{\beta }_{T}\left(T-{T}_{1}\right)+\left(C-{C}_{w}\right)\right]sinsin \left(\alpha \right)-\sigma {B}_{0}^{2}{\Psi }^{\prime},$$

5 $${\left(\rho {C}_{p}\right)}_{f} {\nu }_{0}\frac{dT}{dy}=\frac{d}{dy}\left({k}^{\mathrm{^{\prime}}}\frac{dT}{dy}\right)+\mu \left[\left(1-n\right)+n\left(\frac{\Gamma }{\sqrt{2}}\right)\frac{d{\Psi }^{\mathrm{^{\prime}}}}{dy}\right] {\left(\frac{d{\Psi }^{\mathrm{^{\prime}}}}{dy}\right)}^{2}-\frac{d{q}_{r}}{dy}+\sigma {B}_{0}^{2}{{\Psi }^{\mathrm{^{\prime}}}}^{2}+{\left(\rho {C}_{p}\right)}_{p}\left[{D}_{B}\frac{dT}{dy}\frac{dC}{dy}+\frac{{D}_{T}}{{T}_{1}}\frac{dT}{dy}\right]\frac{dT}{dy},$$

6 $${\vartheta }_{0 }\frac{dC\left(y\right)}{dy}-{D}_{B}\frac{{d}^{2}C\left(y\right)}{d{y}^{2}}-\frac{{D}_{T}}{{T}_{1}}\frac{{d}^{2}T\left(y\right)}{d{y}^{2}}+{\chi }^{\prime}\left(C\left(y\right)-{C}_{w}\left(y\right)\right)=0,$$

The following are the relevant boundary conditions:

7 $${\Psi }^{\prime}=0; \frac{dT}{dy}=0\,\,\,\, at \,\,\,\,y=0,$$

8 $${\Psi }^{\prime}=0; T={T}_{2}+m\frac{dT}{dy} \,\,\,\,at \,\,\,\,y=h,$$

$${\Psi }^{\prime}\rho h=\mu \Psi ; \xi h=y ; {\theta }_{p}{T}_{1}={T}_{2} ; \theta \left({T}_{2}-{T}_{1}\right)=\left(T-{T}_{1}\right) ; \varphi ({C}_{\infty }-{C}_{w})=C-{C}_{w}$$

$${k}^{\prime}=k\left[1+\epsilon \theta \left(\zeta \right)\right].$$

The Eqs. (1), (2), (3), (4), (5), (6), (7) and (8) can be modified as follows by using the above terms. The equations from (9), (10), (11), (12), (13) and (14) correspondingly represent the non-dimensional model problem of Williamson and hyperbolic tangent nanofluids. The Eqs. (15) and (16) represents the corresponding boundary conditions.

9 $$Re\frac{d\Psi }{d\xi }=P+\left[1+ We \frac{d\Psi }{d\xi }\right]\frac{{d}^{2}\Psi }{d{\xi }^{2}}+\left[G{r}_{T}\left({\theta }_{p}-1\right) \theta +G{r}_{C}\varphi \right]sinsin \left(\alpha \right) -{M}^{2}\Psi ,$$

10 $$RePr\frac{d\theta }{d\xi }=\in {\left(\frac{d\theta }{d\xi }\right)}^{2}+\left(1+\in \theta \right)\frac{{d}^{2}\theta }{d{\xi }^{2}}+\frac{EcPr}{({\theta }_{p}-1)} \left\{\left[1+\left(\frac{We}{2}\right) \frac{d\Psi }{d\xi }\right]{\left(\frac{d\Psi }{d\xi }\right)}^{2}+{M}^{2}{\Psi }^{2}\right\}+Rd{\left[\theta \left({\theta }_{p}-1\right)+1\right]}^{2}\left\{3\left({\theta }_{p}-1\right){\left(\frac{d\theta }{d\xi }\right)}^{2}+\left[\theta \left({\theta }_{p}-1\right)+1\right]\frac{{d}^{2}\theta }{d{\xi }^{2}}\right\}+\left[Nt({\theta }_{p}-1)\frac{d\theta }{d\xi }+Nb\frac{d\varphi }{d\xi }\right]Pr\frac{d\theta }{d\xi },$$

11 $$ReSc \frac{d\varphi }{d\xi }-\frac{{d}^{2}\varphi }{d{\xi }^{2}}-\frac{Nt}{Nb}\frac{{d}^{2}\theta }{d{\xi }^{2}}+\chi \varphi =0,$$

12 $$Re\frac{d\Psi }{d\xi }=P+\left[\left(1-n\right)+n We \frac{d\Psi }{d\xi }\right]\frac{{d}^{2}\Psi }{d{\xi }^{2}}+\left[G{r}_{T}\left({\theta }_{p}-1\right) \theta +G{r}_{C}\varphi \right]sinsin \left(\alpha \right) -{M}^{2}\Psi ,$$

13 $$\begin{aligned} RePr\frac{{d\theta }}{{d\xi }} & = \epsilon \left( {\frac{{d\theta }}{{d\xi }}} \right)^{2} + \left( {1 + \epsilon\theta } \right)\frac{{d^{2} \theta }}{{d\xi ^{2} }} + \frac{{EcPr}}{{\left( {\theta _{p} - 1} \right)}}\left\{ {\left[ {\left( {1 - n} \right)\left( {\frac{{d\Psi }}{{d\xi }}} \right)^{2} + n\left( {\frac{{We}}{2}} \right)\left( {\frac{{d\Psi }}{{d\xi }}} \right)^{3} } \right] + M^{2} \Psi ^{2} } \right\} \\ & + Rd\left[ {\theta \left( {\theta _{p} - 1} \right) + 1} \right]^{2} \left\{ {3\left( {\theta _{p} - 1} \right)\left( {\frac{{d\theta }}{{d\xi }}} \right)^{2} + \left[ {\theta \left( {\theta _{p} - 1} \right) + 1} \right]\frac{{d^{2} \theta }}{{d\xi ^{2} }}} \right\} \\ & + \left[ {Nt\left( {\theta _{p} - 1} \right)\frac{{d\theta }}{{d\xi }} + Nb\frac{{d\varphi }}{{d\xi }}} \right]Pr\frac{{d\theta }}{{d\xi }}, \\ \end{aligned}$$

14 $$ReSc \frac{d\varphi }{d\xi }-\frac{{d}^{2}\varphi }{d{\xi }^{2}}-\frac{Nt}{Nb}\frac{{d}^{2}\theta }{d{\xi }^{2}}+\chi \varphi =0,$$

15 $$\Psi =0; \frac{d\theta }{d\xi }=0 at \xi =0,$$

16 $$\Psi =0; \theta =1+\gamma \frac{d\theta }{d\xi } at \xi =1,$$

where $Rd={\frac{16\sigma {T}_{1}}{3{k}^{*}k}}^{3}-$ thermal radiation parameter, $Ec=\frac{{\mu }^{2}}{{\left(\rho {C}_{p}\right)}_{f}{h}^{2}\rho {T}_{1}}-$ Eckert number, $Re=\frac{{\vartheta }_{0}\rho h}{\mu }-$ Reynolds number, $We=\frac{\sqrt{2}\Gamma \mu }{\rho {h}^{2}}-$ Wiesenberger number, $Pr=\frac{\vartheta {\left(\rho {C}_{p}\right)}_{f}}{k}-$ Prandtl number, $G{r}_{C}=\frac{g{\beta }_{C}{\rho }^{2}{h}^{3} \left({C}_{\infty }-{C}_{w}\right)}{{\mu }^{2}}-$ solutal Grashof number, $G{r}_{T}=\frac{g{\beta }_{T}{\rho }^{2}{h}^{3}{T}_{1}}{{\mu }^{2}}-$ thermal Grashof number, $P=-\frac{\rho {h}^{3}}{{\mu }^{2}}\frac{dp}{dx}-$ pressure gradient parameter, $Pe=RePr=\frac{{\left(\rho {C}_{p}\right)}_{f}{\vartheta }_{0}d}{k}-$ Peclet number, $\gamma =\frac{m}{h}-$ temperature jump parameter, $M=\sqrt{\frac{\sigma }{\mu }} {B}_{0}h-$ magnetic parameter, $\tau =\frac{{\left(\rho {C}_{p}\right)}_{p}}{{\left(\rho {C}_{p}\right)}_{f}}-$ heat capacitance ratio, $Nb=\frac{\tau {D}_{B}\left({C}_{\infty }-{C}_{w}\right)}{\vartheta }-$ Brownian motion parameter, $\chi =\frac{{\chi }^{\prime}{h}^{2}}{{D}_{B}}-$ chemical reaction parameter, $Nt=\frac{\tau {D}_{T}}{\vartheta }-$ thermophoresis parameter, $Sc=\frac{\vartheta }{{D}_{B}}-$ Schmidt number.

## Solution methodology

Solving an initial value problem twice, with step sizes 't' and 't/2', is one technique to ensure accuracy. However, for the lower step size, this requires a considerable amount of computation, which must be repeated if the agreement is found to be inadequate. One option to address this problem is to use the Runge–Kutta-Fehlberg 45 method. It includes a procedure for determining whether the correct step size ${\prime}t{\prime}$ is being utilized. Each stage involves making and comparing two different estimates for the solution. The approximation is approved if the two responses are almost identical. When the relative difference between the previously obtained iterative value and the present iterative value matches, within a tolerance of $10^{ - 6}$, the iterative process is terminated. If the two findings disagree by a certain degree of precision, the step size is decreased. If the answers agree to more significant digits than needed, the step size is raised. We performed the computations for many of the previously given parameter values. Every step requires the application of the subsequent six values;

$${m}_{1}=t\, h\left({x}_{m}, {y}_{m}\right),$$

$${m}_{2}=t\, h\left({x}_{m}+\frac{1}{4}t, {y}_{m}+\frac{1}{4}{m}_{1}\right),$$

$${m}_{3}=t\, h\left({x}_{m}+\frac{3}{8}t, {y}_{m}+\frac{3}{32}{m}_{1}+\frac{9}{32}{m}_{2}\right),$$

$${m}_{4}=t\, h\left({x}_{m}+\frac{12}{13}t, {y}_{m}+\frac{1932}{2197}{m}_{1}-\frac{7200}{2197}{m}_{2}+\frac{7296}{2197} {m}_{3}\right),$$

$${m}_{5}=t\, h\left({x}_{m}+t, {y}_{m}+\frac{439}{216}{m}_{1}-8{m}_{2}+\frac{3680}{513}{m}_{3}-\frac{845}{4104}{m}_{4}\right),$$

$${m}_{6}=t\, h\left({x}_{m}+\frac{1}{2}t, {y}_{m}-\frac{8}{27}{m}_{1}+2{m}_{2}-\frac{3544}{2565}{m}_{3}+\frac{1859}{4104}{m}_{4}-\frac{11}{40}{m}_{5}\right).$$

After that, the RK4 method is used to estimate the answer of the IVP.

$${y}_{m+1}={y}_{m}+\frac{25}{216}{m}_{1}+\frac{1408}{2565}{m}_{3}+\frac{2197}{4101}{m}_{4}-\frac{1}{5}{m}_{5}.$$

The RK5 method is used to get a good value for the solution;

$${y}_{m+1}^{\prime}={y}_{m}+\frac{16}{135}{m}_{1}+\frac{6656}{12825} {m}_{3}+\frac{28561}{56430}{m}_{4}-\frac{9}{50}{m}_{5}+\frac{2}{55}{m}_{6}.$$

The process is terminated if $|{y}_{m+1}-{y}_{m+1}^{\prime}|$ is small enough, or the analysis is continued with a smaller step size ${\prime}t{\prime}$ if $|{y}_{m+1}-{y}_{m+1}^{\prime}|$ is not small enough.

## Entropy generation

The concept of entropy as a physical attribute of the thermodynamic system is formulated by the second law of thermodynamics. Energy transfer activities, as well as the motion of nanoliquid molecules, substance mixing, heat exchange and mass transfer phenomena, are primarily responsible for the generation of entropy in the thermodynamic system. The entropy production mathematical model for Williamson and hyperbolic tangent nanofluid are provided in the following expressions (17) and (18) separately (see Mkwizu et al.^41^, Venkateswara et al.^42^ and Abderrahmane et al.^43^)

17 $$E{g}^{*}=\frac{1}{{T}^{2}}\left[k(1+\varepsilon \theta )+\frac{16{\sigma }^{*}{T}^{3}}{3{k}^{*}}\right]{\left(\frac{dT}{dy}\right)}^{2}+\frac{1}{T}\left\{\mu \left[1+\left(\frac{\Gamma }{\sqrt{2}}\right)\frac{d{\Psi }^{\prime}}{dy}\right] {\left(\frac{d{\Psi }^{\prime}}{dy}\right)}^{2}\right\}+\frac{1}{T}\sigma {B}_{0}^{2}{{\Psi }^{\prime}}^{2}+R\frac{{D}_{B}}{{C}_{\infty }}{\left(\frac{dC}{dy}\right)}^{2}+ R\frac{{D}_{B}}{T}\left(\frac{dT}{dy}\right)\left(\frac{dC}{dy}\right),$$

18 $$E{g}^{*}=\frac{1}{{T}^{2}}\left[k(1+\varepsilon \theta )+\frac{16{\sigma }^{*}{T}^{3}}{3{k}^{*}}\right]{\left(\frac{dT}{dy}\right)}^{2}+\frac{1}{T}\left\{\mu \left[\left(1-n\right)+n\left(\frac{\Gamma }{\sqrt{2}}\right)\frac{d{\Psi }^{\prime}}{dy}\right] {\left(\frac{d{\Psi }^{\prime}}{dy}\right)}^{2}\right\}+\frac{1}{T}\sigma {B}_{0}^{2}{{\Psi }^{\prime}}^{2}+R{D}_{B}\left[\frac{1}{{C}_{\infty }}{\left(\frac{dC}{dy}\right)}^{2}+ \frac{1}{T}\left(\frac{dT}{dy}\right)\left(\frac{dC}{dy}\right)\right].$$

Each term on the right-hand side, from the first to fourth, describes the entropy created by heat transfer, viscous dissipation, Joule heating, and mass diffusion in the models above. The non-dimensional form of (17) and (18) are represented by the two equations below.

19 $$Eg=\left\{1+\varepsilon \theta +Rd{\left[\theta \left({\theta }_{p}-1\right)+1\right]}^{3}\right\}\left\{\frac{{\left({\theta }_{p}-1\right)}^{2}}{{\left[\theta \left({\theta }_{p}-1\right)+1\right]}^{2}}\right\}{\left(\frac{d\theta }{d\xi }\right)}^{2}+\frac{Br}{\left[\theta \left({\theta }_{p}-1\right)+1\right]}\left\{{M}^{2}{\Psi }^{2}+\left[1+\left(\frac{We}{2}\right) \frac{d\Psi }{d\xi }\right]{\left(\frac{d\Psi }{d\xi }\right)}^{2}\right\}+\omega {{\omega }^{*}}^{2}{\left(\frac{d\varphi }{d\xi }\right)}^{2}+\omega {\omega }^{*}\left\{\frac{\left({\theta }_{p}-1\right)}{\left[\theta \left({\theta }_{p}-1\right)+1\right]}\right\}\left(\frac{d\theta }{d\xi }\right)\left(\frac{d\varphi }{d\xi }\right),$$

20 $$Eg=\left\{1+\varepsilon \theta +Rd{\left[\theta \left({\theta }_{p}-1\right)+1\right]}^{3}\right\}\left\{\frac{{\left({\theta }_{p}-1\right)}^{2}}{{\left[\theta \left(\zeta \right)\left({\theta }_{p}-1\right)+1\right]}^{2}}\right\}{\left(\frac{d\theta }{d\xi }\right)}^{2}+\frac{Br}{\left[\theta \left(\zeta \right)\left({\theta }_{p}-1\right)+1\right]}\left\{{M}^{2}{\Psi }^{2}+\left[\left(1-n\right){\left(\frac{d\Psi }{d\xi }\right)}^{2}+n\left(\frac{We}{2}\right) {\left(\frac{d\Psi }{d\xi }\right)}^{3}\right]\right\}+\omega {{\omega }^{*}}^{2}{\left(\frac{d\varphi }{d\xi }\right)}^{2}+\omega {\omega }^{*}\left\{\frac{\left({\theta }_{p}-1\right)}{\left[\theta \left({\theta }_{p}-1\right)+1\right]}\right\}\left(\frac{d\theta }{d\xi }\right)\left(\frac{d\varphi }{d\xi }\right).$$

where, $\omega =\frac{R{D}_{B}{C}_{\infty }}{k} -$ diffusive variable, ${\omega }^{*}=\frac{{C}_{w}-{C}_{\infty }}{{C}_{\infty }}-$ characteristic concentration ratio.

$$Bejan \,\,number=\frac{entropy \,\,generation\,\, by \,\,energy \,\,and \,\,solutal \,\,transfer}{total\,\, entropy \,\,generation}.$$

## Results and discussion

The momentum, energy, concentration, entropy generation and Bejan number for both Williamson and tangent hyperbolic nanofluid in an inclined channel with different parameters were compared graphically in this section. The experimental values of the parameters have been set at $Ec=0.5, G{r}_{T}= G{r}_{C}=0.6, K=0.2, M=1.5, Nb=Nt=0.04, Pr=7, Rd=Re=1, Sc=0.15,We=0.3,\alpha =\frac{1}{2}, \epsilon =0.2, \theta =1.5$ throughout the computation. The numerical results are compared with published results in Table 1 and attained a good agreement with each other.

**Table 1:** Comparison of the present numerical findings for $-\theta ^{\prime}(0)$ for some reduced cases.

$\xi$	Eegunjobi1 and Makinde^51^		Present results
	Exact solution	Numerical solution	
$0.9$	$0.046264$	$0.046264$	$0.046265$
$0.8$	$0.079042$	$0.079042$	$0.079043$
$0.7$	$0.099930$	$0.099930$	$0.099931$
$0.6$	$0.110257$	$0.110257$	$0.110258$
$0.5$	$0.111127$	$0.111279$	$0.111279$

**Figure 2:**
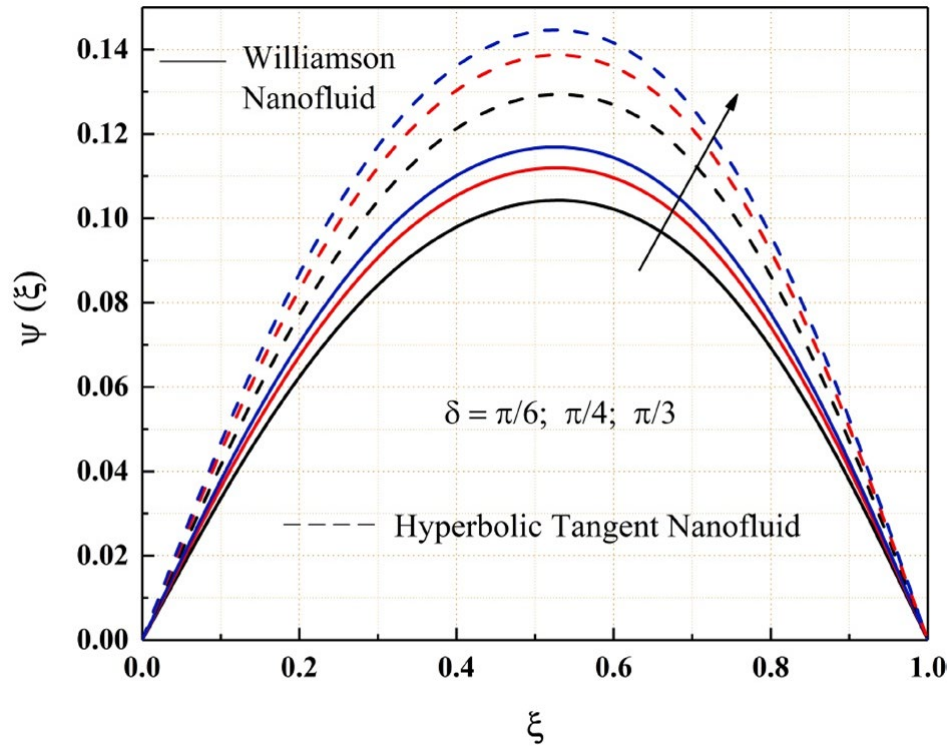
ẟ impact on $$\psi .$$

**Figure 3:**
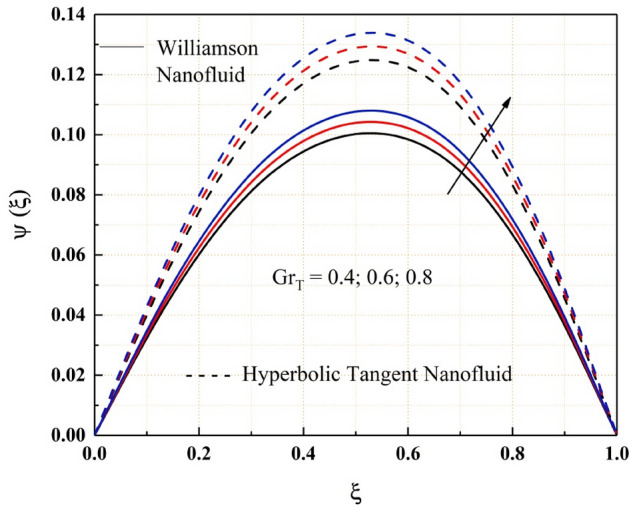
$$G{r}_{T}$$ impact on $$\psi .$$

**Figure 4:**
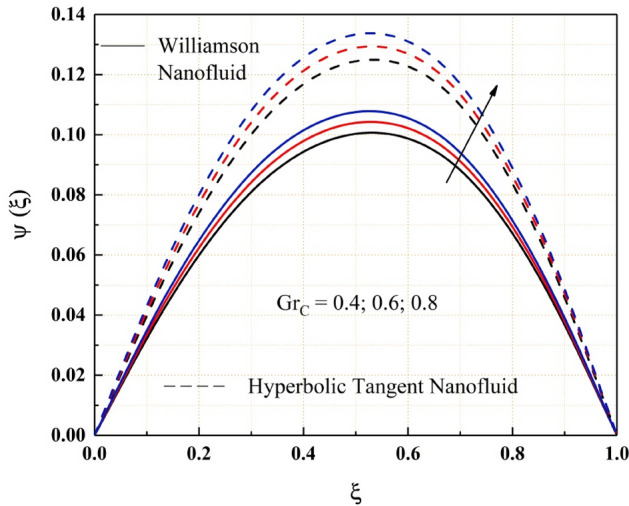
$$G{r}_{C}$$ impact on $$\psi .$$

**Figure 5:**
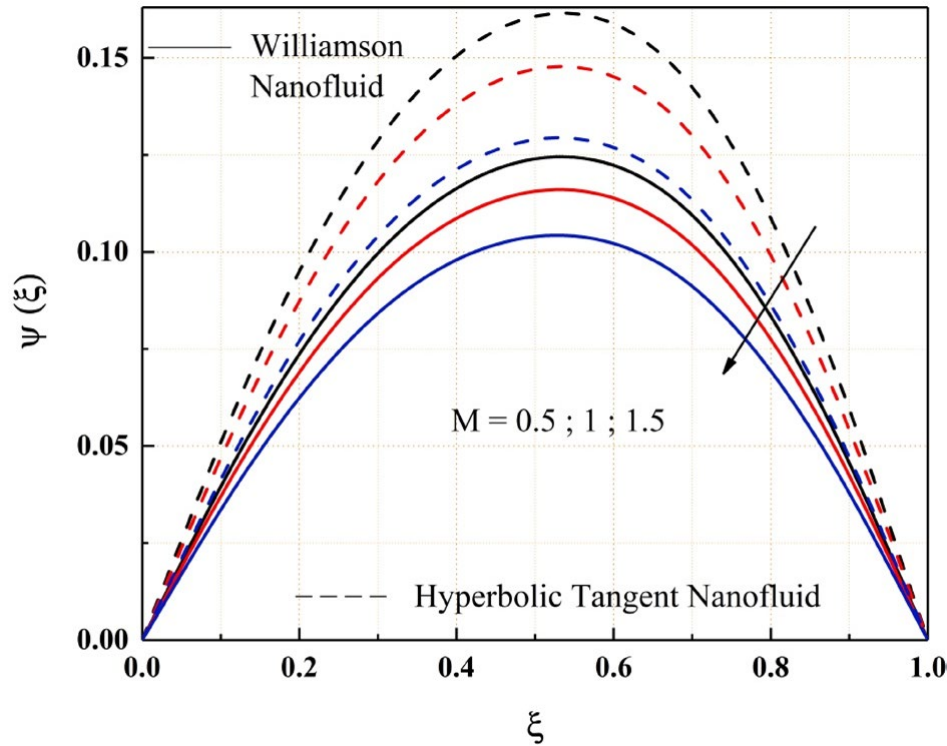
$$M$$ impact on $$\psi .$$

**Figure 6:**
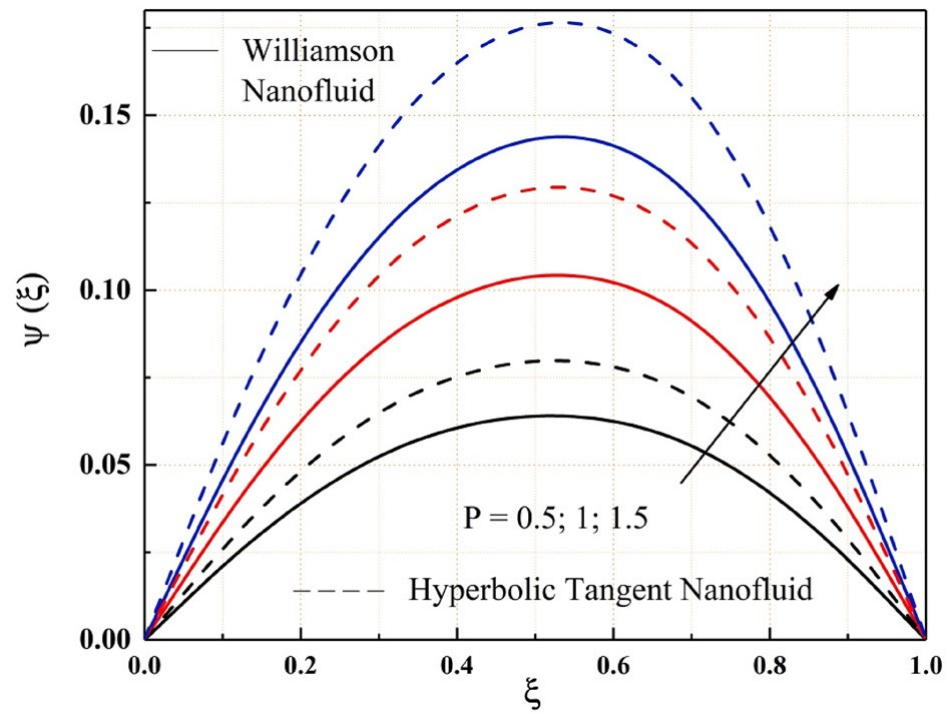
$$P$$ impact on $$\psi .$$

**Figure 7:**
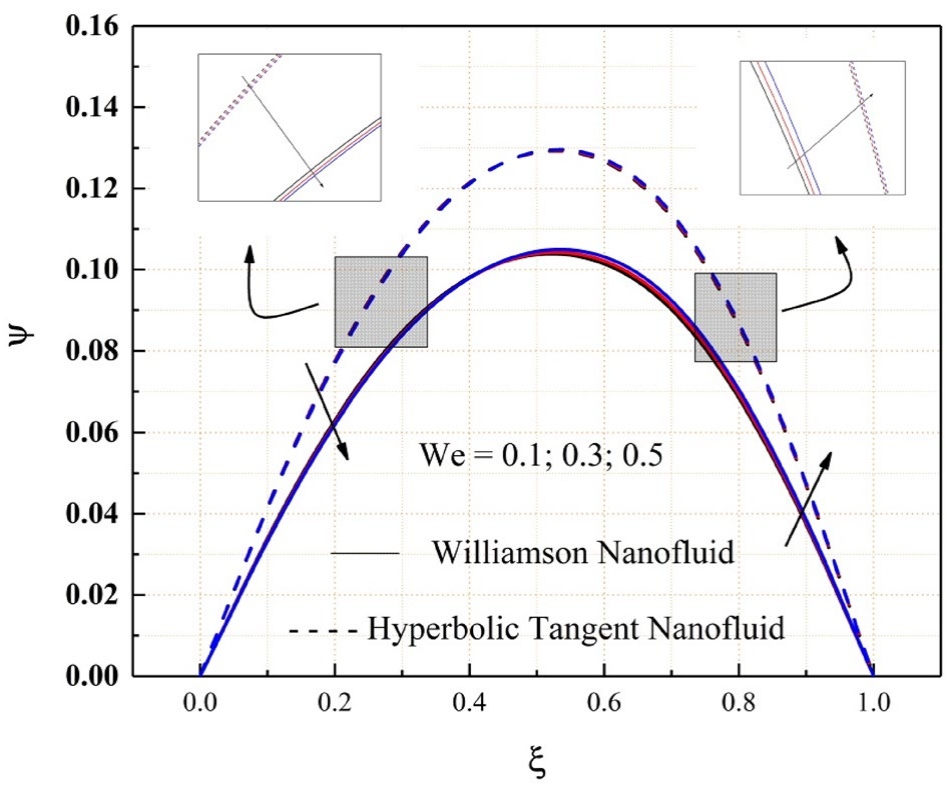
$$We$$ impact on $$\psi .$$

**Figure 8:**
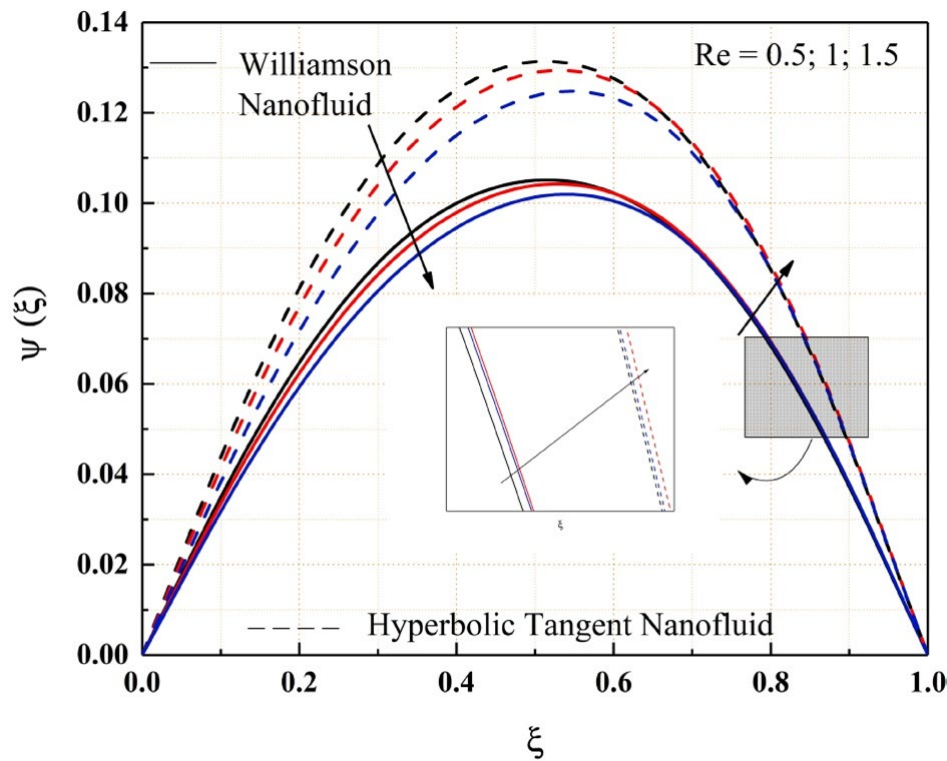
$$Re$$ impact on $$\psi .$$

The Figs. 2, 3, 4, 5, 6, 7 and 8 show how pressure gradient parameter, magnetic parameter, Reynolds number, angle of inclination, temperature and the solutal Grashof number influences on the flow field of Williamson nanofluid and hyperbolic tangent nanofluid. The hyperbolic tangent nanofluid has higher velocity than the Williamson nanofluid, as shown in these figures. More pressure is exerted to the flow system as the angle of inclination increases, resulting in increased Williamson and hyperbolic tangent nanofluid velocity, as illustrated in Fig. 2. The non-Newtonian nanofluids velocity increases when the both the Grashof numbers increase, as seen in Figs. 3 and 4. Because the parameters thermal and solutal Grashof numbers correspondingly enhance the thermal and solutal buoyancy forces. The flow rate of both Williamson and hyperbolic tangent nanofluids is reduced when the magnetic parameter is raised, as seen in Fig. 5. The Lorentz force, which opposes nanofluid flow, is produced by the interaction between transfer magnetic flux and electric field. The influence of the pressure gradient parameter on the nanofluids momentum profile is be seen in Fig. 6. The increased value of the pressure gradient parameter improves the velocity of both Williamson and hyperbolic tangent nanofluids in this graph. Figure 7 displays the effect of the Weissenberg number on the nanofluids' momentum profile. Because both non-Newtonian Williamson and hyperbolic tangent nanofluids possess shear thinning behavior, the velocity profile nature reduces at the bottom plate and increases at the upper plate. The effect of Reynolds number on the velocity profile is seen in the diagram Fig. 8. Due to the supply of heated fluid from the below channel plate and the fluid suck from the above channel plate, fluid velocity drops at the below porous plate and increases at the top porous plate.

**Figure 9:**
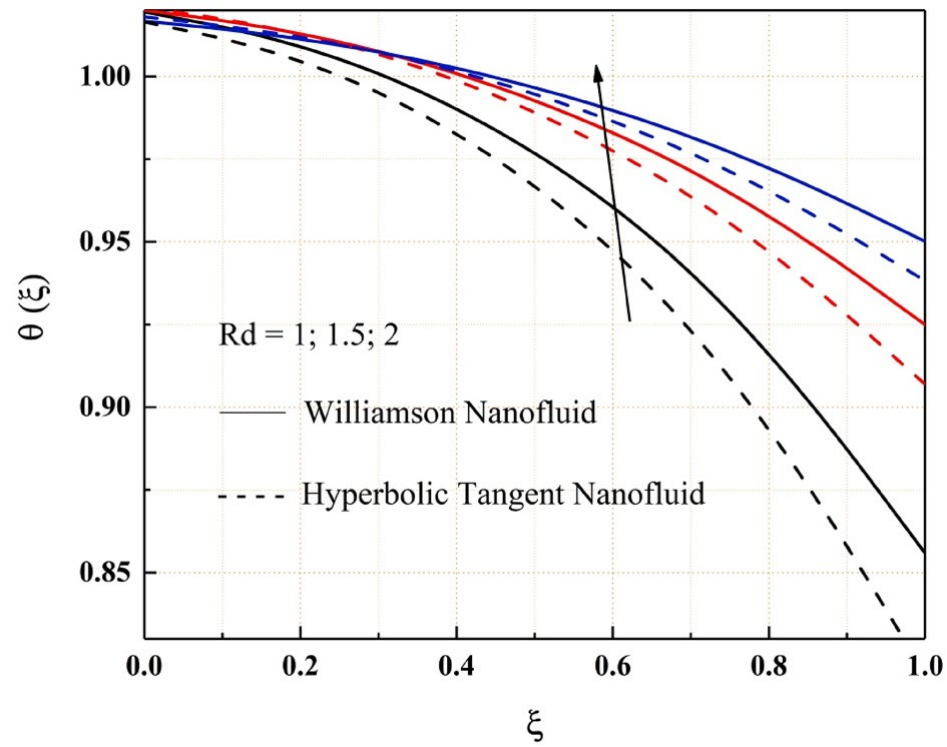
$$Rd$$ impact on $$\theta .$$

**Figure 10:**
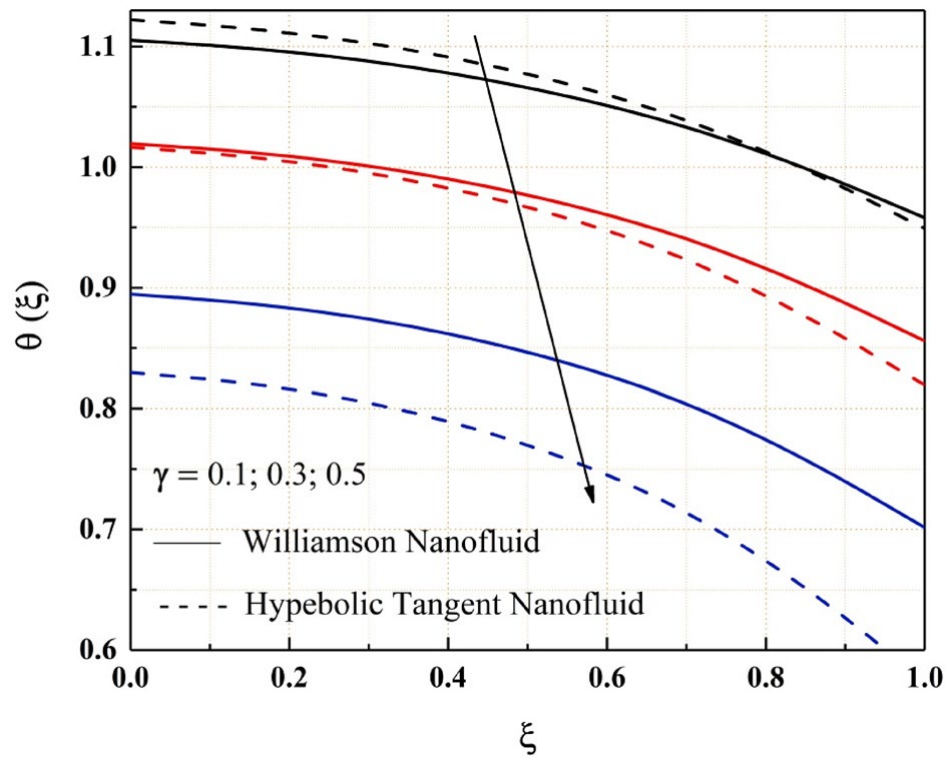
$$\gamma$$ impact on $$\theta .$$

**Figure 11:**
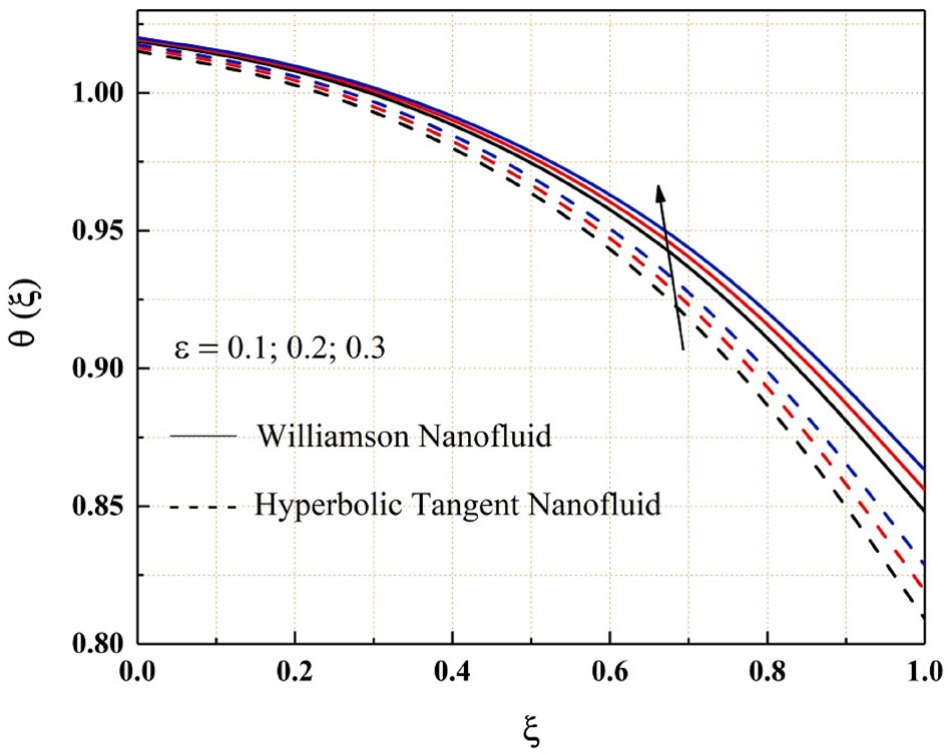
$$\epsilon$$ impact on $$\theta .$$

**Figure 12:**
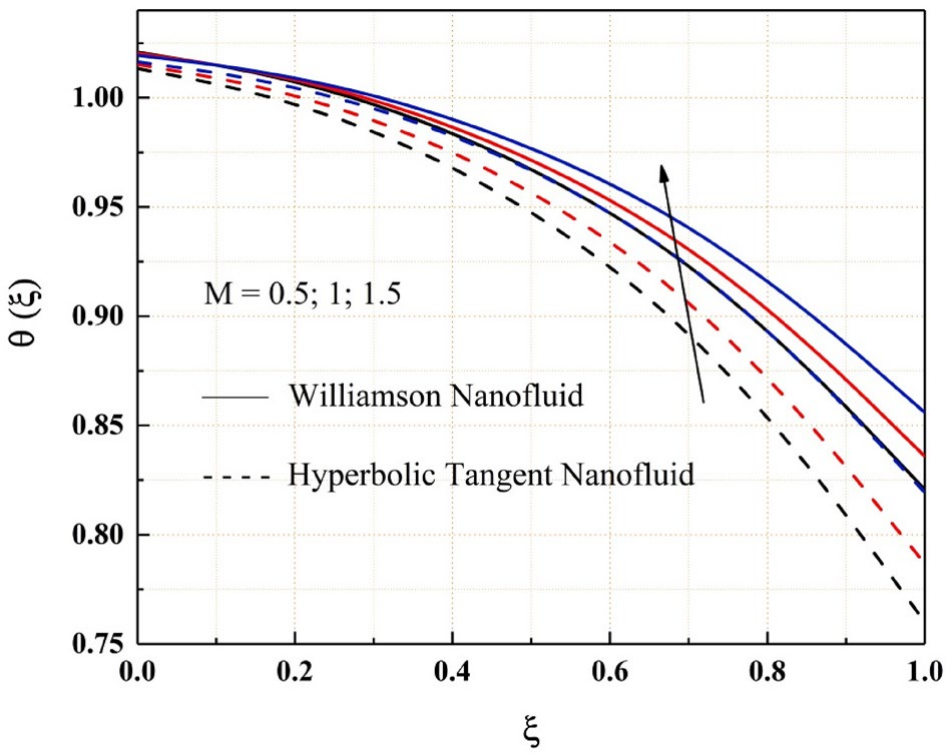
$$M$$ impact on $$\theta .$$

**Figure 13:**
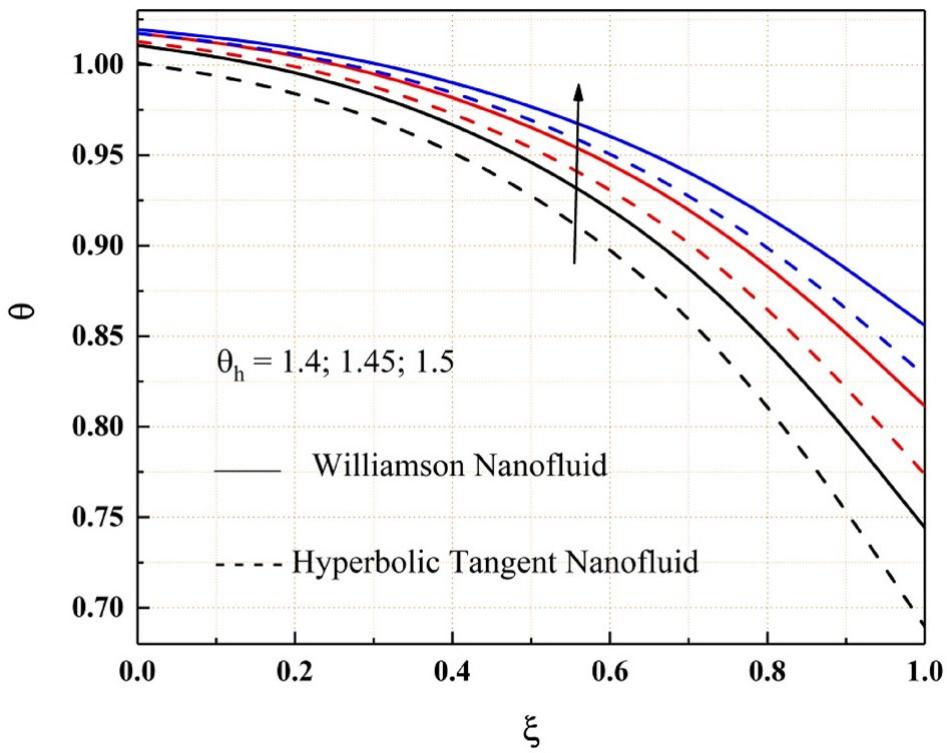
$${\theta }_{h}$$ impact on $$\theta .$$

The consequence of temperature, magnetic, variable thermal conductivity, thermal radiation, and temperature jump parameters on the heat transfer of both Williamson and hyperbolic tangent nanofluid have been remarked from the Figs. 9, 10, 11, 12 and 13. Williamson nanofluid has a higher energy profile than hyperbolic tangent nanofluid in all these thermal field graphs. In the microchannel system, the heat transport rate of hyperbolic tangent nanofluid is better than Williamson nanoliquid. Figure 9 shows how the thermal energy of nanofluids falls as the radiation parameter is increased. One of the heat transfer processes that cools the system is thermal radiation. The heat transfer process is also applicable in this channel system, where a rise in the value of thermal radiation improves the rate of heat transfer, reducing the total thermal energy of the fluid. As the temperature jump parameter is enhanced, the heat transfer rate accelerates, and as a result, the temperature drops, as seen in the Fig. 10. Figures 11 and 12 show how the increased magnitude of variable viscosity and magnetic parameters increases fluid temperatures. Both of these increased values of the parameters decrease the flow rate of the nanoliquid, resulting in a decrease in heat transfer rate, and these magnetic and variable thermal conductivity parameters increase the thermal conduction rate, resulting in an increase in Williamson and hyperbolic tangent nanofluid thermal energy. The temperature of the nanofluids rises as the temperature parameter rises, as seen in Fig. 13. This outcome is owing to the additional energy provided to the system by the temperature parameter.

**Figure 14:**
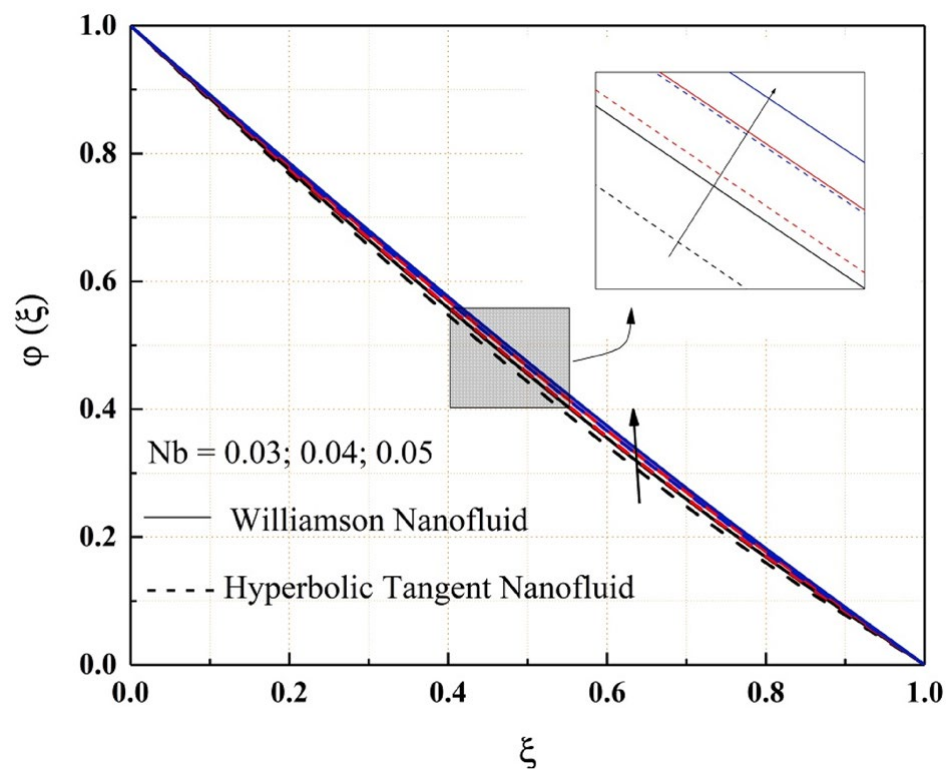
$$Nb$$ impact on $$\varphi .$$

**Figure 15:**
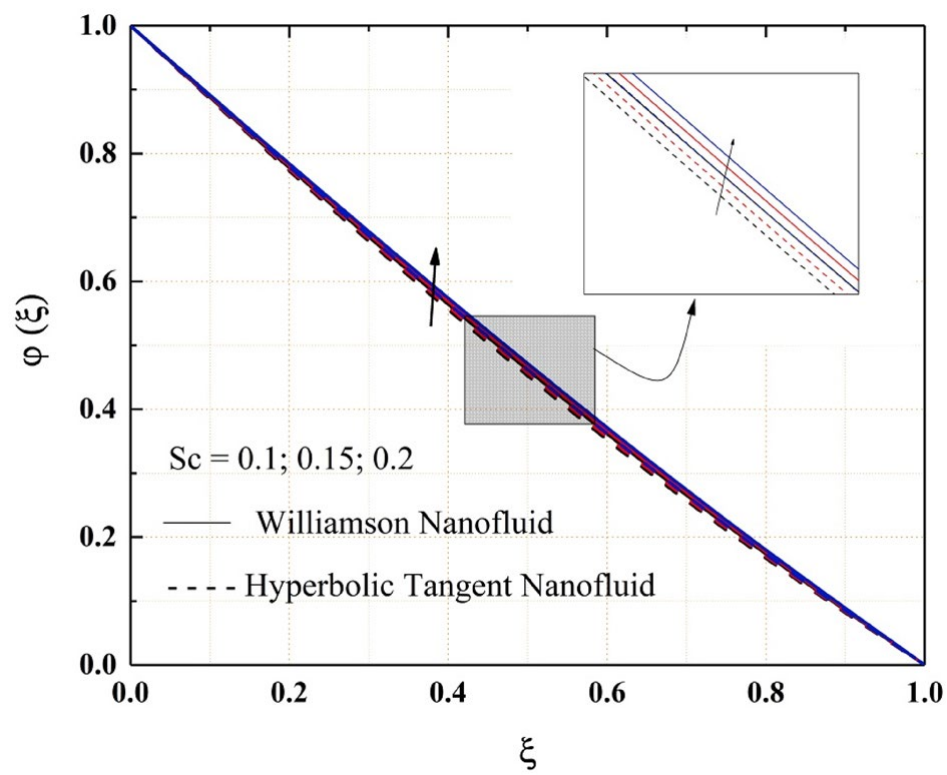
$$Sc$$ impact on $$\varphi .$$

**Figure 16:**
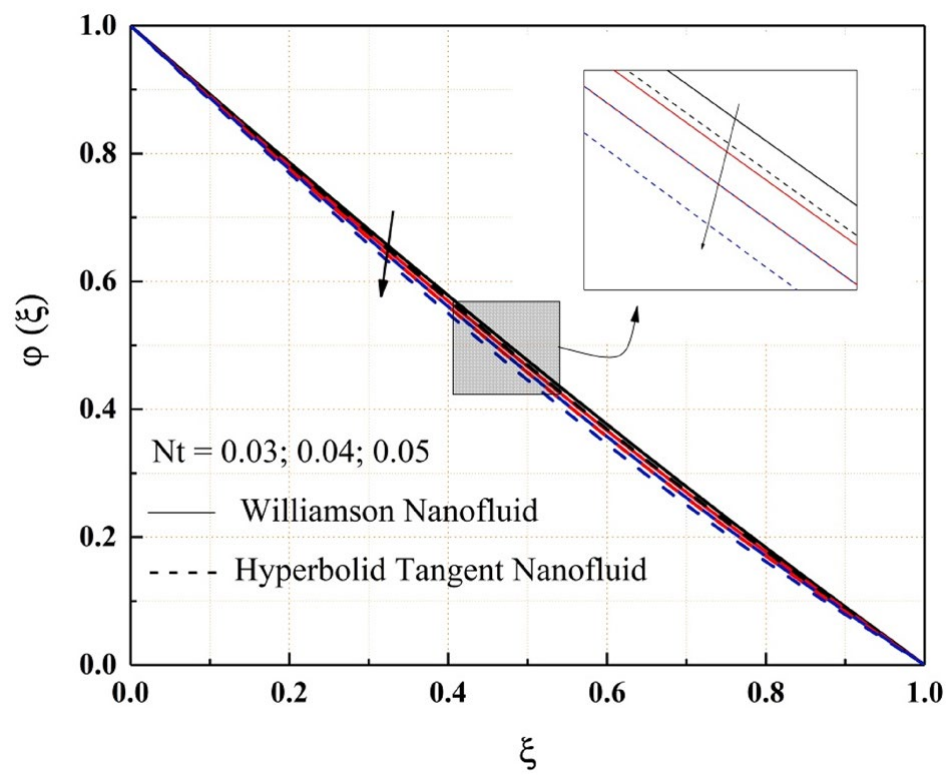
$$Nt$$ impact on $$\varphi .$$

**Figure 17:**
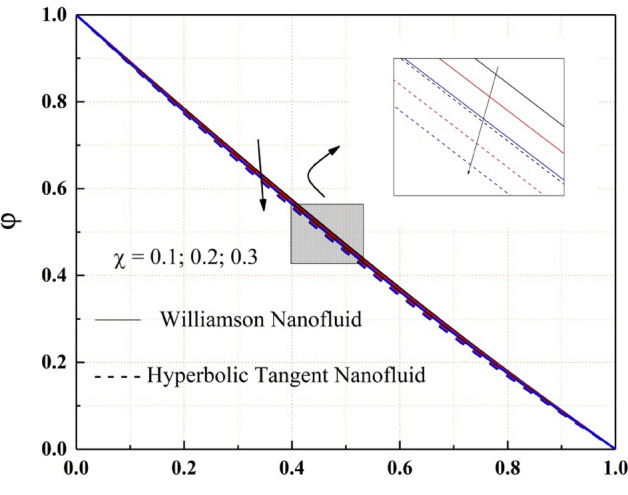
$$\chi$$ impact on $$\varphi .$$

Figures 14, 15, 16 and 17 show how nanofluid concentration changes when Brownian motion, thermophoresis, Schmidt number, and chemical reaction parameter values rise. In these graphs, it's also important to note that the hyperbolic tangent nanofluids concentration is lower than the Williamson nanofluids throughout the analysis. Because of the random mobility of the molecules, the concentration profile of both Williamson and hyperbolic tangent nanofluids increases with Brownian motion parameter and Schmidt number, as seen in Figs. 14 and 15. Volatility is caused by non-compensated collisions with the surrounding molecules. These oscillations are responsible for the diffusion process. Diffusion is the net flow of matter from a high-concentration to a low-concentration region caused by random molecular mobility. The decreasing trend of the concentration profile for a higher value of the thermophoresis parameter and chemical reaction parameter are related to the migration of large or colloidal molecules in the fluid, as seen in Figs. 16 and 17.

**Figure 18:**
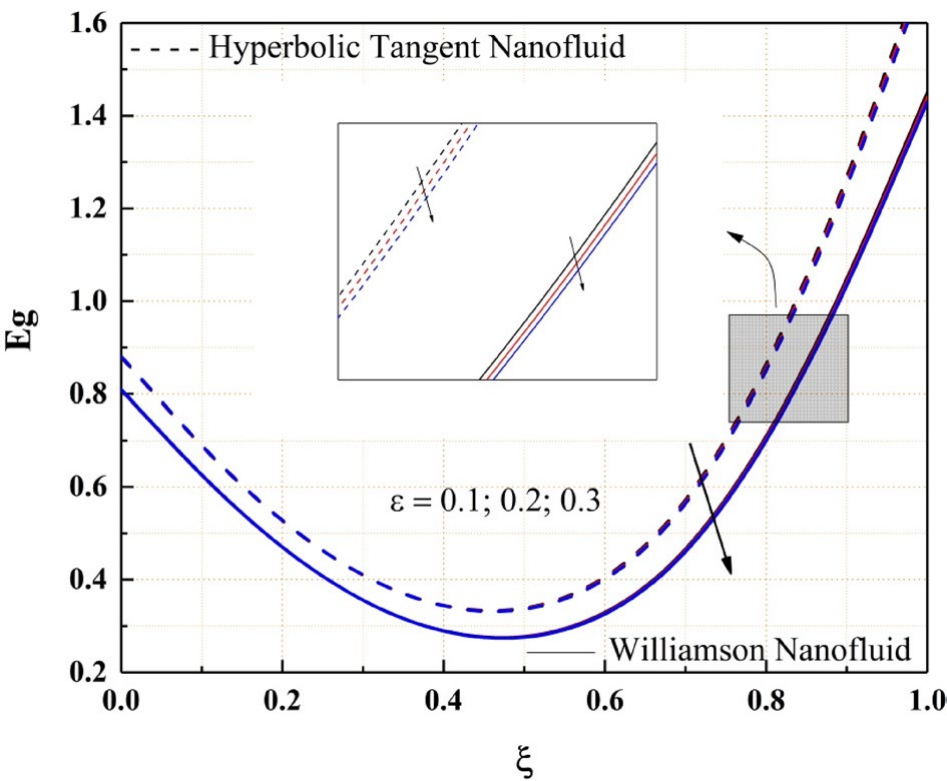
$$\epsilon$$ impact on $$Eg.$$

**Figure 19:**
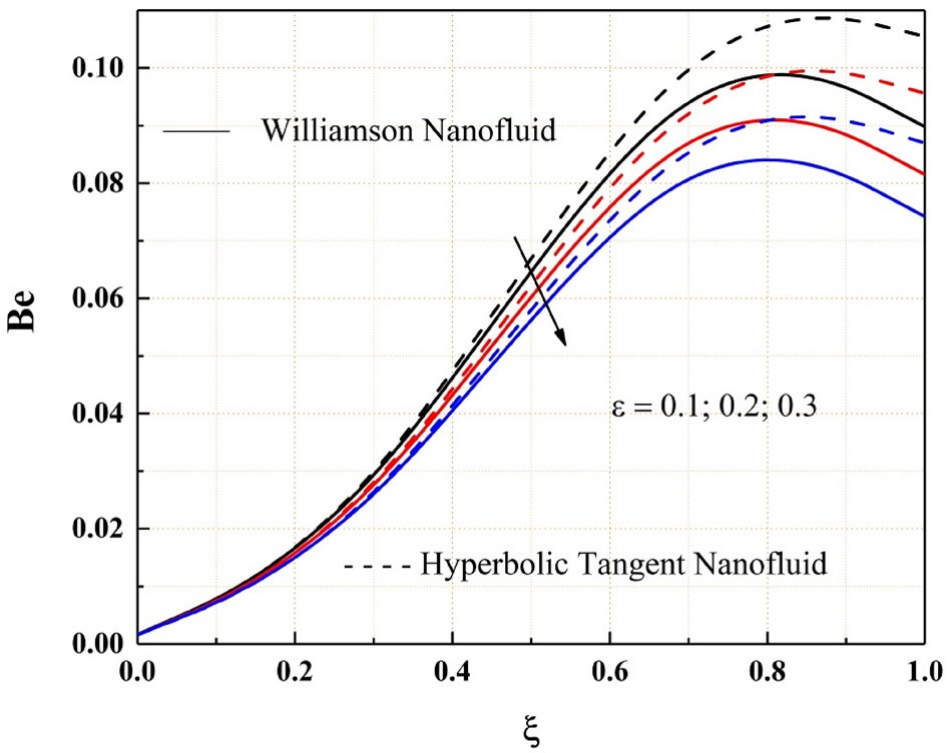
$$\epsilon$$ impact on $$\varphi .$$

**Figure 20:**
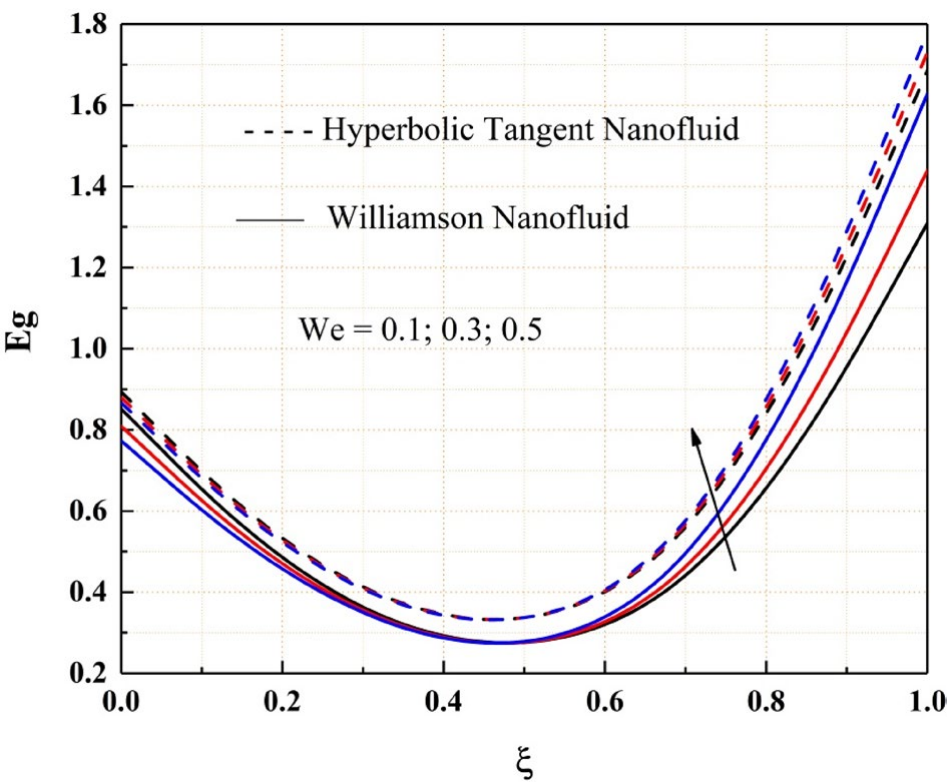
$$We$$ impact on $$Eg.$$

**Figure 21:**
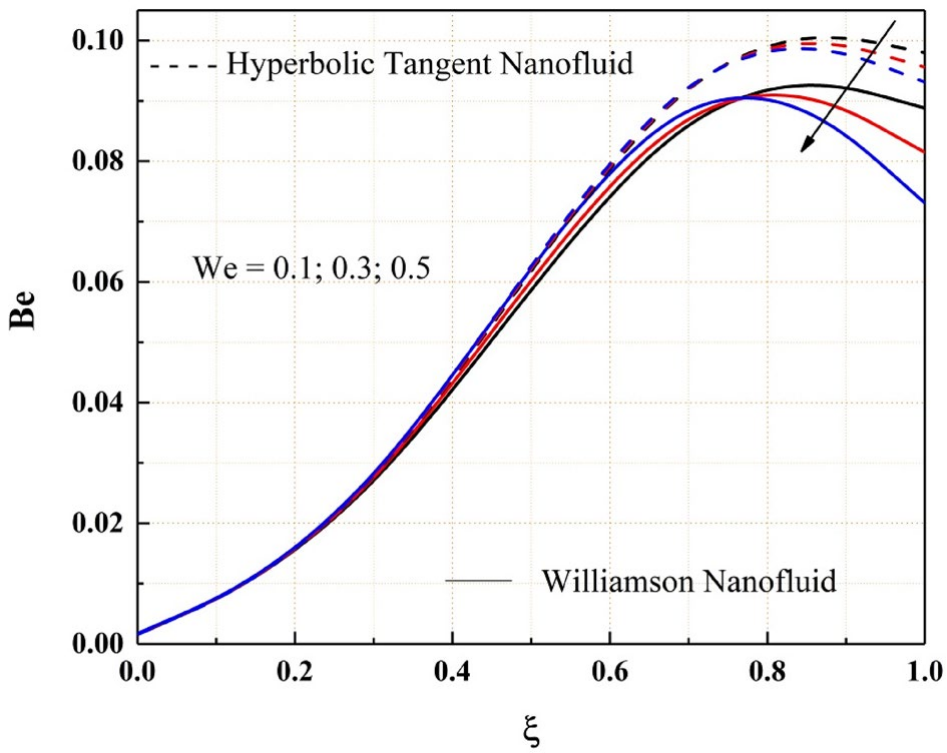
$$We$$ impact on $$Be.$$

As shown in Fig. 18, the entropy generation of the Williamson and hyperbolic tangent nanofluids reduces as the variable thermal conductivity parameter is increased. As a result of the increased values of the parameters, the fluid flow rate lowers, and the flow system's uncertainty diminishes. Also, as seen in Fig. 19, the Bejan number rises as the magnitude of thermal conductivity parameter develops. The entropy generation and Bejan number profile nature with Weissenberg number are described in the Figs. 20 and 21 respectively. Entropy generation grows in the right permeable plate of the channel and drops in the left permeable plate, whereas the Bejan number profile reveals the opposite entropy nature due to the system’s total molecular randomness by viscous dissipation. Also, all the graphs from 18, 19, 20 and 21 demonstrated clearly that the Williamson nanofluid’s total entropy production, including its Bejan number, is less than the hyperbolic tangent nanofluid.

## Conclusions

In this study, the flow of Williamson and hyperbolic tangent shear thinning nanofluids in an inclined microchannel was analyzed separately using numerical methods and the momentum, thermal, concentration fields, and entropy generation rate of these non-Newtonian nanofluids were compared using the graphs. As compared to Williamson nanofluid, hyperbolic tangent nanofluid is high velocity in an inclined channel. The velocity profiles shows parabolic nature that is compared to velocity of the both non-Newtonian nanofluids at the channel plates, velocity is high at core region. The increased values of angle of inclination, pressure gradient parameter and thermal and solutal Grashof number increases the velocity of the nanofluids. But heightened magnitude of $We$ and $Re$ shows dual nature that is at the lower plate velocity decreases and at the above channel plate velocity increases. As magnetic parameter increase, velocity decreases. Temperature of the hyperbolic tangent nanoliquid is lower than Williamson nanofluid. The temperature jump parameter decreases the temperature of the channel system. The thermal radiation, magnetic and temperature parameter heightens the temperature profile. The concentration profile enhances with Brownian motion and Schmidt number while decreases with thermophoresis and chemical reaction parameter. Here noted that to maintain a velocity, temperature and concentration of the nanofluids in the channel the magnetic parameter, temperature jump parameter and thermophoresis parameters are adjusted consequently. Minimization of the system entropy can be achieved by increasing the value of variable thermal conductivity parameter. Irreversibility rate increases at right channel plate and decreases at left channel plate with $We$. Bejan number decrease with all the parameters such as $We and \varepsilon .$ For the various technological and commercial applications, the link between flow, mass, and heat transport properties in nanoliquids with entropy formation is crucial. Entropy formation, convective heat conditions, Stefan blowing, non-uniform heat sources/sinks, and Newtonian heating may all be explored in nanoliquid stream models. In the future, a mathematical model for non-Newtonian liquid flow may be created using an enhanced numerical model for hydrodynamic and thermal interface constraints.

## Data Availability

The datasets used and/or analysed during the current study available from the corresponding author on reasonable request.

## List of symbols

$$p$$: Pressure term, $$(kg\cdot {m}^{-1}\cdot {s}^{-2})$$
$n$: Power law index
$$g$$: Gravitational force**,** $$(m/{s}^{2})$$
$${B}_{0}$$: Magnetic effect**,** $$(tesla )$$
$${C}_{p}$$: Heat capacitance**,** $$(J/kg/K)$$
${k}^{\prime}$: Variable thermal conductivity
$$k$$: Thermal conductivity, $$(W{\cdot m}^{-1}{\cdot K}^{-1})$$
${D}_{B}$: Brownian motion co-efficient
${D}_{T}$: Thermophoresis co-efficient
$T$: Temperature of the fluid**,** $(K)$
$${T}_{1}$$: Ambience temperature**,** $$(K)$$
${T}_{2}$: Injected hot fluid temperature
$$C$$: Solutal concentration**,** $$(Kg/{m}^{3})$$
${C}_{w}$: Concentration at the channel wall, $(Kg/{m}^{3})$
$Rd$: Thermal radiation parameter
$$Ec$$: Eckert number
$$M$$: Magnetic parameter
$$Re$$: Reynolds number
$$We$$: Wiesenberger number
$$Pr$$: Prandtl number
$G{r}_{C}$: Solutal Grashof number
$$Eg$$: Entropy generation
$$Be$$: Bejan number
$$Sc$$: Schmidt number
$$Nt$$: Thermophoresis parameter
$Nb$: Brownian motion parameter
$G{r}_{T}$: Thermal Grashof number
$P$: Pressure gradient parameter
$$Pe$$: PECLET number

## Greek symbols

${\nu }_{0}$: Fluid suction/injection**,** $(m/s)$
$$\psi$$: Dimensionless velocity
$\theta$: Dimensionless thermal energy
$${\psi }^{\prime}$$: Axial velocity, $$(m/s)$$
$\varphi$: Dimensionless fluid concentration
$$\mu$$: Dynamic viscosity**,** $$(Kg {m}^{-1}{s}^{-1})$$
$$\rho$$: Density**,** $$(kg\cdot {m}^{-3})$$
${\beta }_{T}$: Thermal expansion co-efficient**,** $\left({K}^{-1}\right)$
${\beta }_{C}$: Solutal expansion co-efficient**,** $\left({mol}^{-1}\right)$
${\chi }^{\prime}$: Chemical reaction co-efficient
$\epsilon$: Variable thermal conductivity parameter
$\chi$: Chemical reaction parameter
$$\omega$$: Diffusive variable
${\omega }^{*}$: Characteristic concentration ratio
$\gamma$: Temperature jump parameter
$\tau$: Heat capacitance ratio
$$\sigma$$: Electrical conductivity**,** $$(S\cdot {m}^{-1})$$
$$\Gamma$$: Time relaxation**,** $$({s}^{-1})$$
$\alpha$: Angle of inclination
